# LXRα limits TGFβ-dependent hepatocellular carcinoma associated fibroblast differentiation

**DOI:** 10.1038/s41389-019-0140-4

**Published:** 2019-05-16

**Authors:** Anita Morén, Claudia Bellomo, Yutaro Tsubakihara, Dimitris Kardassis, Wolfgang Mikulits, Carl-Henrik Heldin, Aristidis Moustakas

**Affiliations:** 1 Department of Medical Biochemistry and Microbiology, Science for Life Laboratory, Uppsala University, Box 582, Biomedical Center, SE-751 23 Uppsala, Sweden; 2 Ludwig Institute for Cancer Research, Uppsala University, Box 595, Biomedical Center, SE-751 24 Uppsala, Sweden; 3 Department of Biochemistry, University of Crete Medical School, 71003 Heraklion, Crete Greece; 4 Department of Medicine I, Division: Institute of Cancer Research, Comprehensive Cancer Center Vienna, Medical University of Vienna, Vienna, Austria

**Keywords:** Cancer, Cell biology, Molecular biology

## Abstract

Transforming growth factor β (TGFβ) is deposited in the extracellular space of diverse tissues. Resident fibroblasts respond to TGFβ and undergo myofibroblastic differentiation during tissue wound healing and cancer progression. Cancer-associated fibroblasts (CAFs) communicate with tumor cells during cancer progression, under the guidance of TGFβ signaling. We report that agonist-activated liver X receptors (LXR) limit the expression of key components of myofibroblast differentiation, including the *α-smooth muscle actin* (*αSMA*) gene in liver cancer cells. CAFs derived from hepatocellular carcinoma (HCC) express high αSMA and low LXRα levels, whereas hepatocarcinoma cells exhibit an inverse expression pattern. All hepatoma cells analyzed responded to the LXRα agonist T0901317 by inducing *fatty acid synthase* (*FASN*) expression. On the other hand, T0901317 antagonized TGFβ-induced fibroblastic marker responses, such as fibronectin and calponin, in a subset of hepatoma cells and all CAFs analyzed. Mechanistically, LXRα antagonized TGFβ signaling at the transcriptional level. Smad3 and LXRα were recruited to adjacent DNA motifs of the *ACTA2* promoter. Upon cloning the human *ACTA2* promoter, we confirmed its transcriptional induction by TGFβ stimulation, and LXRα overexpression repressed the promoter activity. Hepatosphere formation by HCC cells was enhanced upon co-culturing with CAFs. T0901317 suppressed the positive effects exerted on hepatosphere growth by CAFs. Taken together, the data suggest that LXRα agonists limit TGFβ-dependent CAF differentiation, potentially limiting primary HCC growth.

## Introduction

Misregulation of transforming growth factor β (TGFβ) signaling occurs in infectious, cardiovascular diseases and cancer^1^. TGFβ halts cell proliferation and accelerates cell death, as it occurs in immune cells or during liver homeostasis^2,3^. Moreover, TGFβ promotes epithelial-mesenchymal transition (EMT) and stimulates fibroblasts to secrete cytokines, chemokines and extracellular matrix (ECM) molecules^2,3^. Cellular responses initiate when TGFβ binds to the type II (TβRII) and type I (TβRI) kinase receptor complex^4^. Within this complex, TβRII trans-phosphorylates TβRI, activating TβRI to phosphorylate Smad2 and Smad3, which, in complex with Smad4, regulate gene transcription. The TGFβ receptor also activates mitogen activated protein kinase (MAPK), the tyrosine kinase Src and phosphatidylinositol-3´-kinase pathways. Upon ligand-induced cleavage, the TβRI intracellular domain regulates transcription^4,5^.

In adult tissues, TGFβ regulates EMT and fibroblast maturation^6,7^. Transcriptional programs become activated in epithelial cells by TGFβ, mediated for example by *Snail* and *Slug*, leading to repression of cell-cell contact genes, induction of cytokine genes as well as ECM genes, such as fibronectin^8^. EMT generates mesenchymal-like cells that partially resemble fibroblasts and contribute to chronic tissue fibrosis and cancer progression^9^. Cancer-associated fibroblasts (CAFs) secrete TGFβ and other cytokines to assist tumor cell invasion and suppress anti-tumor immune responses^10,11^. Myofibroblast differentiation is a key response to TGFβ signaling, mediated by Smad complexes with β-catenin and parallel MAPK signaling^6,12^. The contractility of CAFs depends on microfilament networks built by α smooth muscle actin (αSMA) and associated myosins, tethered via membrane receptors to specialized collagen fibers, all induced by TGFβ signaling^6,12^.

Among several malignancies, TGFβ regulates the progression of hepatocellular carcinoma (HCC), a cancer with high-mortality rate worldwide^13,14^. TGFβ suppresses HCC by arresting hepatocyte proliferation at the early G1 phase of the cell cycle, and inducing apoptotic responses^13,14^. TGFβ promotes EMT, survival and CAF differentiation, contributing to HCC invasion and metastasis^14–16^. Similar to TGFβ, the nuclear liver X receptors (LXRα/NR1H3 and LXRβ/NR1H2), either suppress or promote cancer, by inhibiting cell proliferation or helping tumor cells to escape from immune detection, respectively^17^. The LXRs are expressed in hepatocytes among other tissues; agonistic LXR ligands include the oxysterols, metabolic derivatives of cholesterol, inducing the transcriptional activity of LXRs^18^.

We previously demonstrated that LXRα suppresses TGFβ-induced differentiation^19^, and, in the context of HCC, we described a role for the transcription factor Snail as a target of the crosstalk between TGFβ and LXRα^20^. Due to the established role of TGFβ on CAF biology, we here analyzed signaling crosstalk between TGFβ and LXRα in human HCC fibroblasts

## Results

### LXRα expression is enriched in epithelial, whereas the TGFβ-induced myofibroblast gene *αSMA* is enriched in mesenchymal, HCCs

**Fig. 1:**
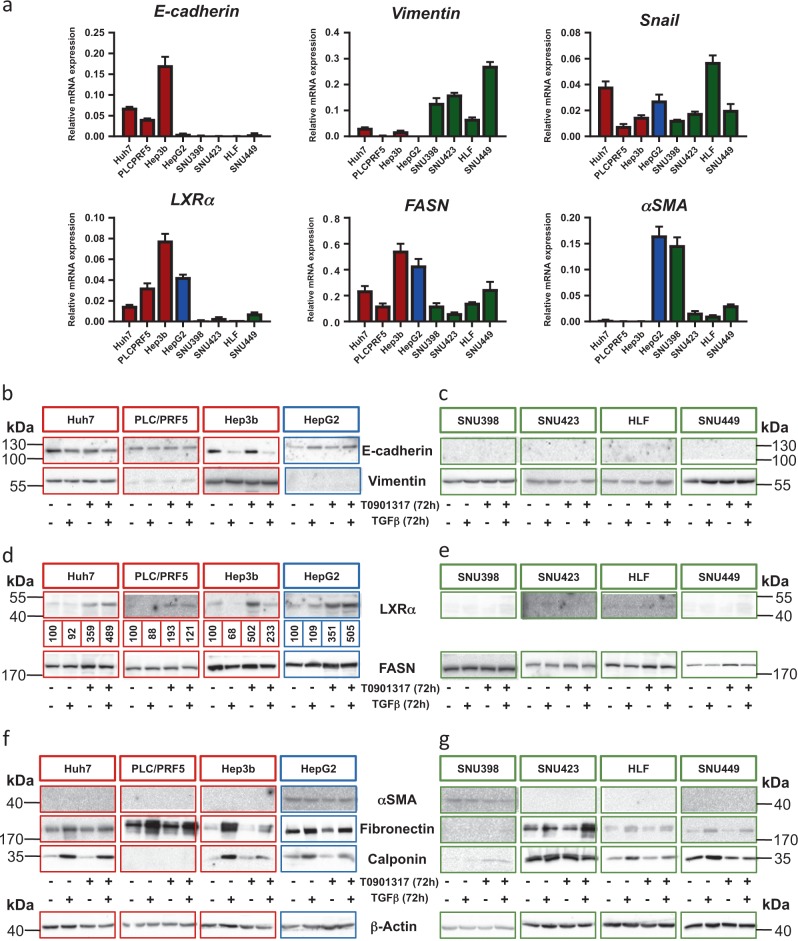
LXRα expression is enriched in epithelial human liver cancer cells. Human HCC cells were cultured and the expression of several genes and proteins was assessed. **a** mRNA expression was assessed via real-time PCR and normalized to the expression of *GAPDH*. Mean ± SD values are plotted. Experiments were performed in biological triplicates (*n*_*b*_ = 3), each of them in technical triplicate (*n*_*t*_ = 3). **b**–**g** The indicated human HCC cells were stimulated with DMSO (Mock), 5 ng/ml TGFβ1, 5 µM T0901317 or a combination of the last two for 72 h prior to protein immunoblot. The β-actin immunoblot serves as loading control for all panels (**b**–**g**). Experiments were performed in biological triplicates (*n*_*b*_ = 3). Densitometric values of LXRa protein are also included. Molecular size markers are indicated in kDa. Graph bars and immunoblot images are color-marked: epithelial (red), HepG2-mixed phenotype (blue), mesenchymal (green)

We screened HCC cell lines previously classified based on their response to TGFβ signaling, as epithelial-like cells with an early TGFβ target gene signature (Huh7, PLC/PRF5, Hep3b, HepG2) and as mesenchymal-like cells with a late signature (SNU398, SNU423, HLF, SNU449)^21^. Using E-cadherin (epithelial) and vimentin (mesenchymal) as marker genes, we found good concordance of mRNA and protein expression profiles with the classification (Fig. 1a–c), but also detected vimentin mRNA and protein expression in Huh7 and Hep3b cells (Fig. 1a–c). *Snail* mRNA expression was used as an intermediate mediator of mesenchymal differentiation, and was found to be expressed by most HCC cells (Fig. 1a).

All epithelial HCC cells expressed high, whereas the mesenchymal HCC cells expressed low, LXRα mRNA and protein levels (Fig. 1a, d, e). Fatty acid synthase (*FASN*) mRNA, a direct readout of LXRα transcriptional activity^17^, had a rather similar to the *LXRα* mRNA expression profile (Fig. 1a). The response of the HCC cells to the well-established LXR agonist, T0901317^17^, was measured by analyzing FASN protein and mRNA expression. The epithelial HCCs expressed endogenous FASN protein (Fig. 1d), reflecting the mRNA profile (Fig. 1a, Suppl. Fig. S1), and T0901317 stimulation induced FASN in all epithelial HCCs examined, which was easier to detect at the mRNA (Suppl. Fig. S1) than at the protein level (Fig. 1d). As previously reported^19,20^, T0901317 stimulation enhanced LXRα levels by 2-5-fold in epithelial HCCs (Fig. 1d densitometry). TGFβ stimulation had no appreciable effect on FASN expression, and combination of TGFβ with T0901317 normalized *FASN* levels to basal in Huh7, Hep3b and HepG2 cells (Fig. 1d). LXRα stabilization appeared somewhat reduced after co-treating the cells with TGFβ and T0901317, but only in Hep3b cells (Fig. 1d).

In the mesenchymal HCCs SNU398, SNU423, HLF and SNU449, basal LXRα protein expression was at the limit of detection, and T0901317 stimulation did enhance LXRα levels so that they became detectable (Fig. 1e). Accordingly, the mesenchymal HCCs expressed basally endogenous FASN (Fig. 1a, Suppl. Fig. S1) and T0901317 stimulation induced *FASN* mRNA levels to a comparable degree as in epithelial HCC cells (Suppl. Fig. S1); this effect appeared weaker when FASN protein levels were measured (Fig. 1e). Combination of TGFβ and T0901317 stimulation resulted in relatively weaker induction of FASN by T0901317 in two, but not the other, mesenchymal HCCs examined (Fig. 1e, Suppl. Fig. S1). The data indicate that many HCC models respond to LXRα agonist and TGFβ stimulation; in certain HCCs, TGFβ partially antagonizes the stimulatory effect of T0901317 on FASN expression and LXRα stabilization.

### Activation of LXRα suppresses myofibroblastic genes induced by TGFβ

αSMA represents a hallmark gene of activated fibroblasts^6,12^; in contrast to the *LXRα* expression profile, *αSMA* levels were low and high in epithelial and mesenchymal HCCs, respectively (Fig. 1a). Only HepG2 cells expressed high *LXRα*, *FASN* and *αSMA* mRNA levels (Fig. 1a, blue bar differentiates HepG2 from other HCC cells). In agreement with the mRNA profiles, only HepG2 and SNU398 cells expressed αSMA protein, whose levels only slightly changed upon TGFβ or T0901317 stimulation (Fig. 1f, g).

We also examined fibronectin and calponin expression, as additional readouts of TGFβ response and fibroblast activation (Fig. 1f, g). In epithelial HCC cells, TGFβ induced fibronectin (all cells tested) and calponin (all cells except PLC/PRF5) and T0901317 reduced this response primarily in Hep3b cells (Fig. 1f). In the mesenchymal HCC cells (except SNU398), TGFβ induced fibronectin whereas T0901317 did not exhibit any appreciable effect (Fig. 1g); TGFβ also induced calponin and T0901317 normalized the induction to basal level (Fig. 1g). Our observations suggest that T0901317 can suppress TGFβ-induced myofibroblastic markers in about 50% of the HCC cell models.

**Fig. 2:**
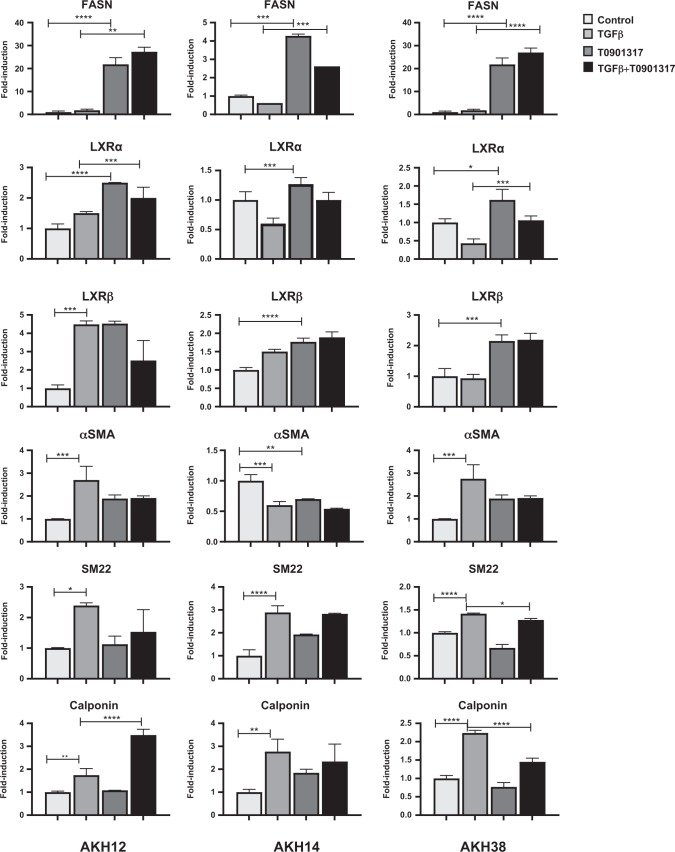
The LXRα agonist T0901317 suppresses TGFβ-induced myofibroblast gene expression in CAFs. AKH12, AKH14 and AKH38 CAFs were treated in complete medium for 74 h; TGFβ1 (5 ng/ml) was administered with or without LXR agonist (5 μM T0901317). Real-time PCR analysis of the indicated gene expression normalized to the expression of *GAPDH*. Mean ± SD values are plotted. Experiments were performed in biological triplicates (*n*_*b*_ = 3), each of them in technical triplicate (*n*_*t*_ = 3). Statistical comparison (two-sided *t*-test) indicates significant differences, *p < 0.05; **p < 0.01; ***p < 0.001; ****p < 0.0001

We then examined recently isolated human HCC primary CAFs (AKH12, AKH14 and AKH38)^22,23^. All three CAF models responded to T0901317, as revealed by *FASN* induction (Fig. 2). The CAFs expressed detectable *LXRα* and *LXRβ* mRNA levels. TGFβ stimulation enhanced *LXRβ* mRNA expression only in AKH12 cells (Fig. 2). Myofibroblast gene profiling indicated that *αSMA* (AKH14 being an exception), *SM22α* and *calponin* mRNA expression were induced by TGFβ; in AKH38 cells, T0901317 suppressed the TGFβ effects, whereas suppression of αSMA was measurable but not significant in AKH12 cells, where *calponin* expression was even enhanced after co-stimulation (Fig. 2).

**Fig. 3:**
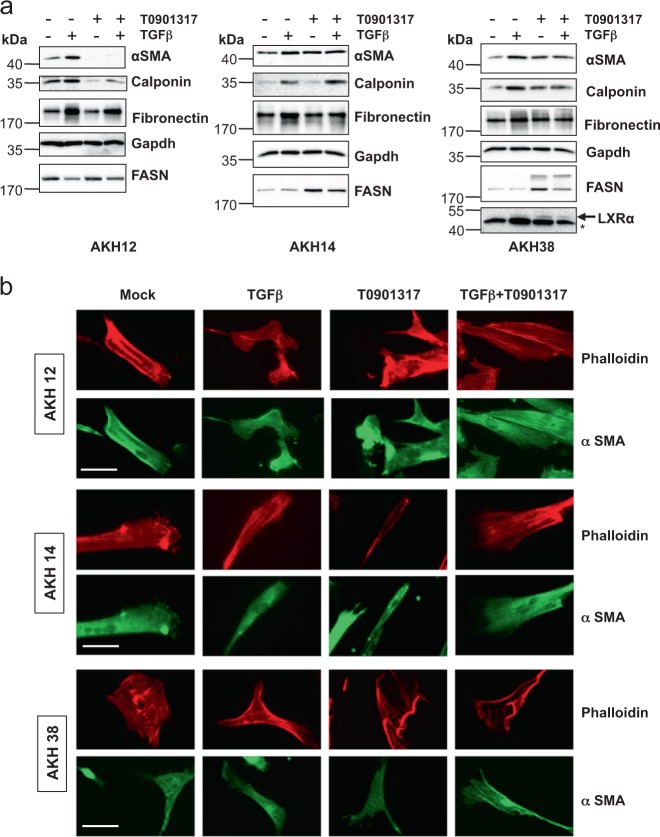
The LXRα agonist T0901317 limits TGFβ-induced myofibroblast differentiation in CAFs. AKH12, AKH14 and AKH38 CAFs were treated in complete medium for 74 h; TGFβ1 (5 ng/ml) was administered with or without LXR agonist (5 μM T0901317). **a** Expression of the indicated proteins was determined by immunoblotting. Experiments were performed in biological duplicates (*n*_*b*_ = 2). An arrow marks the correct protein band and a star a non-specific protein recognized by the LXRα antibody. Molecular size markers are indicated in kDa. **b** F-actin (red) and α-SMA (green) microfilaments and DAPI (blue) in CAFs, after 48 h of the indicated treatments. Bars represent 10 µm. Experiments were performed in biological triplicates (*n*_*b*_ = 3)

The mRNA expression analysis was corroborated by analysis of the corresponding proteins (Fig. 3a). Induction of αSMA, calponin and fibronectin by TGFβ was clearly suppressed in the presence of T0901317 in AKH12 CAFs, less potently in AKH38 CAFs, but not in AKH14 cells (Fig. 3a). FASN protein analysis in the CAF models responding to T0901317 stimulation confirmed the mRNA data (Fig. 2), and TGFβ suppressed the positive effect of T0901317 on FASN (Fig. 3a). The very low endogenous LXRα protein levels in primary CAFs were difficult to assess, but in AKH38 cells, stabilization due to T0901317 stimulation made LXRα detectable (Fig. 3a).

Microscopic analysis of actin-based cytoskeletal organization confirmed abundant accumulation of stress fibers containing β-actin and αSMA and formation of actin-supported membrane ruffles in the three CAFs (Fig. 3b). CAFs stimulated with TGFβ, T0901317 or both exhibited similar phenotype (Fig. 3b). The gene and protein expression data support the responsiveness of patient-derived HCC CAFs to the LXRα agonist T0901317, and an antagonistic effect of LXRα signaling against TGFβ-mediated myofibroblast gene expression in certain HCC CAFs.

### TGFβ and LXRα regulate the human *αSMA*/*ACTA2* gene promoter

**Fig. 4:**
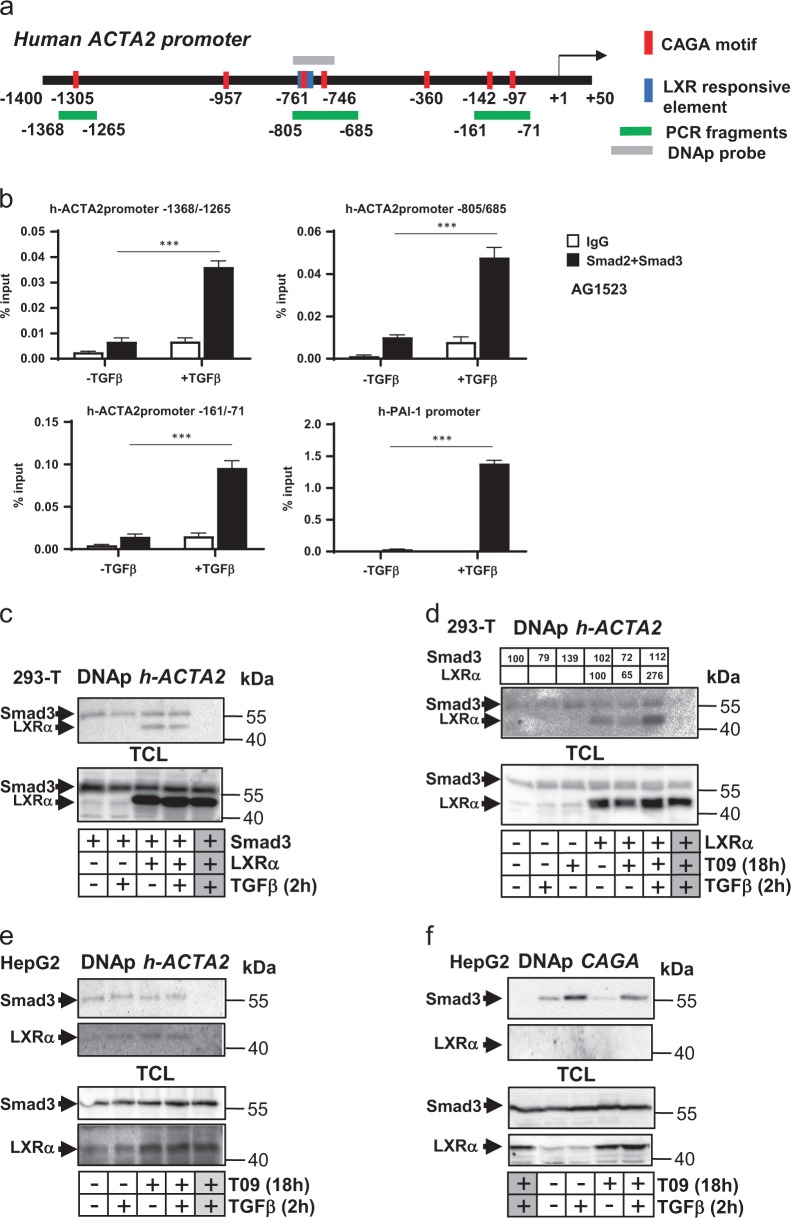
Smads and LXRα bind to the *α-SMA* gene promoter. **a** Diagram of the *hACTA2* gene promoter indicating the transcriptional start site (arrow, +1), putative Smad- (CAGA motif, red boxes) and LXR-binding sites (blue boxes) on the promoter, with numbering relative to the start site corresponding to the first base pair of each CAGA motif and the ends of the promoter fragment. Promoter fragments detected by ChIP (green) and used for DNAp (grey) are shown. **b** ChIP-qPCR analysis of endogenous Smad2/3 binding to three different fragments of the *hACTA2* gene promoter as indicated in (**a**), or to the human *PAI-1* gene, used as positive control. Mean ± SD values are plotted. Experiments were performed in biological duplicates (*n*_*b*_ = 2), each of them in technical triplicate (*n*_*t*_ = 3). Statistical comparison (two-sided *t*-test) indicates significant differences, ***p < 0.001. **c** DNA-precipitation (DNAp) assays using an *hACTA2* gene promoter-specific oligonucleotide and human 293-T cell lysates after transient transfection with pCDNA3-Flag-Smad3 and pCMX-LXRα in the absence (−) or presence (+) of stimulation with 5 ng/ml TGFβ1 for 2 h followed by sequential immunoblotting for transfected Smad3 and LXRα. **d** DNAp using lysate from human 293-T cells transfected only with pCMX-LXRα and stimulated with 5 ng/ml TGFβ1 for 2 h and/or 5 µM T0901317 (T09) for 16 h followed by sequential immunoblotting for endogenous Smad3 and transfected LXRα. Densitometric quantification of the Smad3 and LXRα protein bands is shown. **e** DNAp using lysate from untransfected HepG2 cells stimulated with 5 ng/ml TGFβ1 for 2 h and/or 5 µM T0901317 (T09) for 18 h followed by sequential immunoblotting for endogenous Smad3 and endogenous LXRα. **f** DNAp using a multimerized Smad-binding element (CAGA) oligonucleotide as positive control followed by sequential immunoblotting for endogenous Smad3 and LXRα. Total cell lysates (TCL) indicate expression of each protein, relevant proteins are marked with an arrow, molecular size markers are indicated in kDa, and grey boxes with a plus sign mark control DNAp with streptavidin beads in the absence of specific oligonucleotide to indicate non-specific, background precipitation. Experiments were performed in biological duplicates (*n*_*b*_ = 2), each of them in technical triplicate (*n*_*t*_ = 3)

We examined a possible mechanism by which LXRα signaling might regulate the myofibroblastic response to TGFβ by focusing on the *αSMA*/*ACTA2* gene. Since previous studies of *ACTA2* gene regulation focused on mouse or rat cells, we cloned a fragment of the human *ACTA2* gene spanning the transcriptional start site, extending from −1400 to +50 bp relative to the start site (Fig. 4a).

Bioinformatic analysis of the promoter sequence identified putative TGFβ/Smad-binding elements (CAGA motifs), and LXR-responsive elements (Fig. 4a). ChIP experiments in the HCC CAFs could not succeed due to the low number of primary CAFs available, and primary human foreskin AG1523 fibroblasts were chosen as a good approximation based on established antagonism between TGFβ and LXRα in this cell type^19^. ChIP assays in AG1523 fibroblasts revealed specific binding of Smad2/Smad3 to three adjacent regions of the *ACTA2* promoter, containing CAGA motifs (Fig. 4b). As a positive control, the *plasminogen activator inhibitor 1* (*PAI-1*) gene promoter, a well-characterized target of TGFβ signaling, was analyzed under the same conditions (Fig. 4b). ChIP assays for LXRα could not generate positive results due to the insufficient quality of our antibodies.

In order to bypass the above deficiency and examine binding of LXRα on the *ACTA2* promoter, we analyzed direct binding of Smad3 and LXRα to a synthetic human *ACTA2* gene promoter DNA fragment, which encompasses a cluster of CAGA and LXRα motifs (Fig. 4a). Using protein extracts from human embryonic kidney 293-T (Fig. 4c, d) or human HCC HepG2 cells (Fig. 4e), we detected binding of both Smad3 and LXRα. When Smad3 and LXRα were overexpressed (Fig. 4c), or when only LXRα was overexpressed and Smad3 was endogenous (Fig. 4d), TGFβ or T0901317 stimulation did not affect significantly their binding to the *ACTA2* fragment (Fig. 4d, quantification). In HepG2 HCC cells, endogenous Smad3 and LXRα binding to the *αSMA*/*ACTA2* fragment was recorded, and stimulation with TGFβ, T01901317 alone or together did not significantly affect this binding (Fig. 4e). As positive control for robust TGFβ-dependent Smad3 binding to DNA, a concatameric CAGA DNA fragment was used (Fig. 4f), demonstrating binding in agreement with the PAI-1 promoter ChIP data of Fig. 4b.

**Fig. 5:**
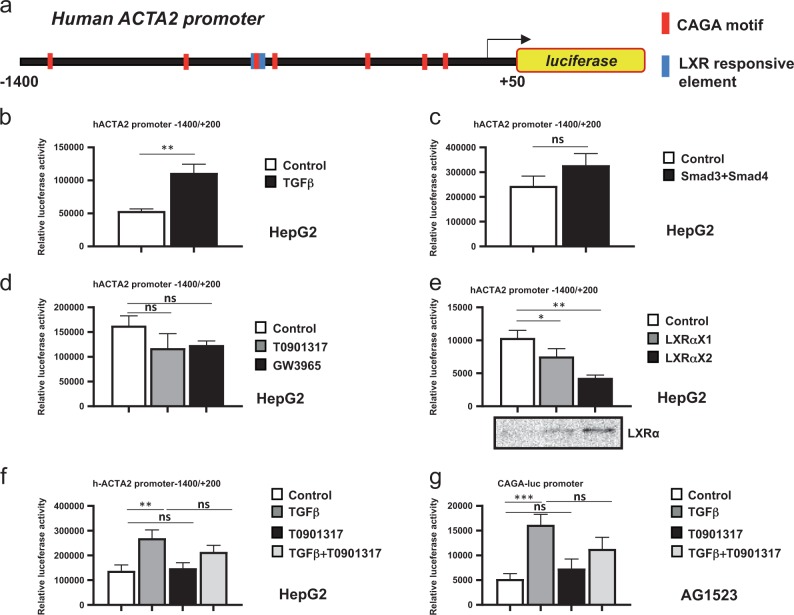
TGFβ induces and LXRα represses *α-SMA* gene promoter activity. **a** Molecular cloning of the *hACTA2* gene promoter, with similar diagram as in Fig. 4a, including the luciferase reporter. **b**–**f** HepG2 cells were simultaneously transfected for 48 h with *hACTA2*-luc reporter and β-gal plasmid alone (**b**, **d**, **f**) or together with empty vector pCDNA3 or pCDNA3-Flag-Smad3 and pCDNA3-Flag-Smad4 (**c**) or pCMX-LXRα full length (**e**). Cells were treated in starvation medium with 5 ng/ml TGFβ1 for 16 h or with T0901317 (5 μM), GW3965 (5 µM) or combination of TGFβ and T0901317 for 16 h, prior to luciferase and β-galactosidase activity detection. **g** Primary human foreskin AG1523 fibroblasts were transfected as above with the *CAGA*-luc reporter and β-gal plasmid followed by treatment in starvation medium with vehicle (control), 5 ng/ml TGFβ1, T0901317 (5 μM) or combination of the last two for 16 h, prior to luciferase and β-galactosidase activity detection. Mean ± SD values are plotted. Experiments were performed in biological triplicates (*n*_*b*_ = 3), each of them in technical quadruplicate (*n*_*t*_ = 4). Statistical comparison (two-sided *t*-test) indicates significant differences, *p < 0.05; **p < 0.01; ***p < 0.001; *ns* *p* > 0.05. Immunoblotting for detection of co-transfected LXRα is provided as control (**e**)

Transient transfection experiments of the *ACTA2* gene promoter-luciferase construct (Fig. 5a) in HepG2 cells revealed its inducible activation by TGFβ stimulation (Fig. 5b). Smad3 and Smad4 co-expression also exhibited a trend of inducing this promoter, but did not score significant (Fig. 5c). The *ACTA2* promoter exhibited a trend for reduced activity in the presence of two different LXRα agonists, T0901317 and GW3965 (Fig. 5d), whereas upon LXRα overexpression at two different concentrations, the promoter activity was reproducibly and more significantly repressed (Fig. 4e). Combining TGFβ and T0901317 stimulation resulted in the same negative trend relative to the positive effect of TGFβ stimulation, yet the repressive effect of LXRα was not significant (Fig. 5f). We also analyzed the CAGA_12_-luciferase promoter, a synthetic promoter that potently responds to TGFβ/Smad signaling^24^. In the AG1523 fibroblasts, as in HCC, the synthetic promoter was activated by TGFβ, whereas T0901317 co-administration exhibited a trend to diminish the promoter activation, which was not significant (Fig. 5g). Thus, increasing LXRα expression causes a reduction of the *ACTA2* promoter activity. All observations support a hypothesis whereby LXRα may negatively regulate the *αSMA*/*ACTA2* gene promoter.

### HCC CAFs promote hepatosphere growth, which is reduced upon LXRα activation

**Fig. 6:**
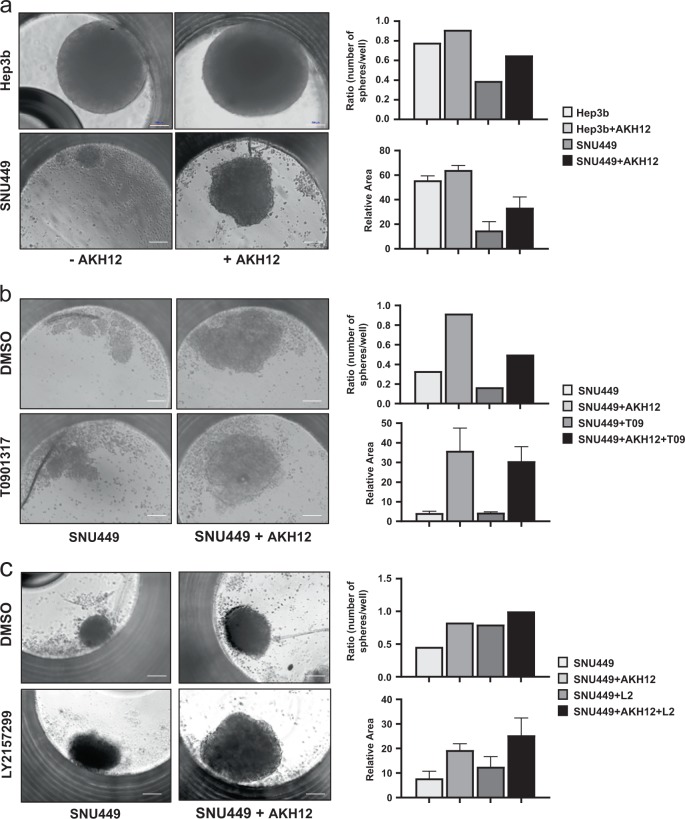
HCC CAFs promote and LXRα activation inhibits 3D hepatosphere formation. AKH12 fibroblasts (14%) and SNU449 or Hep3b hepatoma cells (86%) were co-cultured in hanging drops to generate heterotypic 3D cell populations. **a**–**c** Phase contrast microscopy of individual hepatospheres (left panels, magnification bar, 10 µm). Quantification of hepatosphere cultures (right panels), plotted as mean ± SD values. **a** Plain co-culture and hepatosphere analysis; experiments were performed in biological quadruplicates (*n*_*b*_ = 4). **b** Hepatosphere analysis after stimulation of the AKH12 fibroblasts with T0901317 (5 µM, T09); experiments were performed in biological triplicates (*n*_*b*_ = 3). **c** Hepatosphere analysis after treatment of the co-culture with the TGFβ type I receptor kinase inhibitor LY2157299 (L2, 2 µM); experiments were performed in biological quadruplicates (*n*_*b*_ = 4)

CAFs contribute to tumor progression in response to TGFβ signaling^10,11^ and during HCC development^15,16^. In this context, the role of LXRα remains unexplored. In order to simulate tumor tissue organization, we employed 3D culture conditions that lead to the development of tumor spheroids (hepatospheres) and examined the role of CAFs and LXRα signaling. Hep3b cells assembled typical hepatospheres, while SNU449 formed multicellular aggregations (Fig. 6a). Co-culture with AKH12 CAFs in a cell-to-cell ratio of 1:3 or 1:6 (AKH12 to HCC cells), resulted in larger Hep3b hepatospheres, which formed with higher frequency; AKH12 co-culture with SNU449 showed a more pronounced phenotype as these co-cultures generated architecturally well-organized hepatospheres that formed with higher frequency relative to the SNU449 cells cultured alone (Fig. 6a).

We pretreated AKH12 CAFs with T0901317 for 24 h prior to co-culture with HCC cells, leading to reduced hepatosphere size and number (Fig. 6b). As a specificity control, we pretreated CAFs with a chemical inhibitor of the TGFβ receptor type I kinase (LY2157299), which caused a small increase in hepatosphere size or number (Fig. 6c). Thus, activation of LXRα suppressed 3D hepatosphere growth that was positively promoted by co-culturing CAFs and mesenchymal HCCs.

## Discussion

**Fig. 7:**
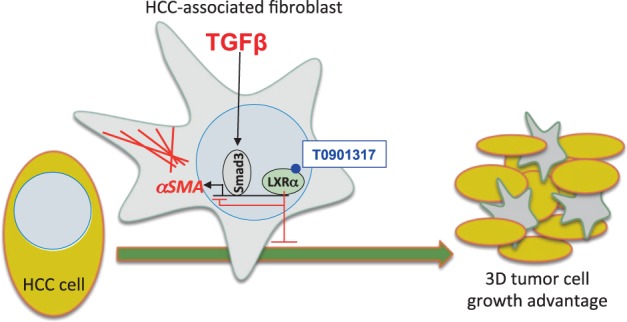
TGFβ and LXR crosstalk between parenchymal and CAF cells in HCC. A liver cancer fibroblast responding to TGFβ by activating Smad3 and inducing α-SMA expression and microfilaments is shown. LXRα binds to the *ACTA2* promoter and upon T0901317 ligand biding, it limits (red negative arrow) the α-SMA response to TGFβ. The CAF together with HCC cells promotes 3D tumor cell growth, which is counteracted (red negative arrow) by the action of T0901317

Based on an unbiased screen for chemical compounds aimed to block TGFβ-induced EMT, we previously explored mechanisms by which oxysterols, the physiological ligands of the LXR transcription factors, antagonize a subset of responses to TGFβ signaling^19^. LXR agonists also block Snail-dependent mesenchymal differentiation, survival mechanisms and Snail-independent pro-apoptotic responses to TGF-β^20^. By focusing on HCC models, we now established that such antagonism regulates CAF responses to TGFβ signaling, at least in part via a gene regulatory mechanism whereby LXRα limits the TGFβ inducibility of target genes, such as *αSMA/ACTA2* (Figs. 1–5). A correlate of this antagonism in the context of liver cancer is the ability of LXRα agonists to limit the growth and expansion of HCC 3D spheroids, a phenotype positively regulated by CAFs when co-cultivated with HCC cells (Fig. 6). We propose that oxysterols acting on HCC CAFs have the ability to down-modulate responses to TGFβ, which impacts on HCC survival and proliferation (Fig. 7).

A majority of epithelial HCC cells expressed detectable LXRα levels and responded to the LXR agonist T0901317 (Fig. 1d, Suppl. Fig. S1), whereas mesenchymal-like HCC cells and HCC CAFs, expressed weakly detectable LXRα levels, and responded only weakly to T0901317 stimulation (Figs. 1e, 2, 3a, Suppl. Fig. S1). Our recent study suggests that LXRα signaling counteracts TGFβ-mediated Snail expression^20^; independent in vivo studies corroborate the above, since in the *LXRα*^−/−^ mouse model, high Snail expression levels were measured in the prostate epithelium^25^. Furthermore, studies in A549 lung adenocarcinoma cells revealed that oxysterol signaling via LXRα/β promoted Snail expression^26^. Thus, in the context of cancer, the impact of LXRα signaling needs to be evaluated with a specific focus on the diverse cellular components of the tumor microenvironment. In the present study, we propose that LXRα signaling limits the TGFβ-mediated mesenchymal and fibroblast differentiation.

Smad3 and LXRα recognize distinct CAGA and LXRE DNA motifs, respectively^5,17^, which are encompassed in the cloned *αSMA/ACTA2* promoter (Fig. 4a). By analyzing the regulation of the *αSMA/ACTA2* gene (Figs. 4 and 5) we provided evidence that LXRα can associate with the *αSMA/ACTA2* promoter; possibly, such binding limits the accessibility of the promoter to TGFβ/Smad3-mediated co-activators (Fig. 7). Previous work showing a synergy between Smad2, Smad3 and LXR signaling via the co-activator protein RAP250^27^ does not explain the observations obtained in HCCs. In several HCC cells, regulation of additional myofibroblastic genes, including *calponin*, *SM22α* and *fibronectin* by LXRα was evident (Figs. 1, 2, 3a). It is therefore possible that LXRα may exert a more general mechanism counteracting TGFβ-mediated differentiation responses.

HCC and CAF 3D co-culture assays were employed in order to simulate an in vivo representative biological context (Fig. 6). Epithelial Hep3b cells with an early TGFβ gene signature^21,23^, generated spheroids independent of the presence of CAFs. Mesenchymal SNU449 HCCs with a late TGFβ gene signature, proposed to express stem cell genes^21,23^, formed “disorganized hepatospheroids” (Fig. 6). Unexpectedly, SNU449-CAF co-culture positively influenced the growth and size of the hepatospheroids (Fig. 6), suggesting that CAFs promote the acquisition of a more regular organoid architecture. The positive trophic and architectural effect of AKH12 CAFs over the SNU449 HCC cells in forming hepatospheres, was reduced by administration of T0901317 only to the CAF population (Fig. 6). This suggested that possible mediators produced by CAFs, which promote hepatosphere architecture and growth, are repressed by LXRα signaling. This mediator generated by CAFs may not be TGFβ itself (Fig. 6c), although TGFβ is important in colon cancer CAFs^11^ and in HCC stellate cells^15^. Further analysis about the signaling pathways, CAF-secreted cytokines and/or cell-cell contact proteins are required to address this important aspect. It should also be noted that the primary patient-derived CAFs exhibit to some extent patient-specific expression profiles and responses to signaling molecules. Thus, we propose that oxysterols provide anti-TGFβ inputs via LXRα, which limit myofibroblastic differentiation and communication between CAFs and carcinoma cells in liver tumors.

## Materials and methods

### Cell culture and treatments

Hep3b, HepG2, PLC/PRF5, Huh7, SNU449, SNU398, SNU423, HLF, HEK-293T (abbreviated as 293-T), HaCaT and AG1523 cells were previously reported^19,28,29^, and maintained in (Hep3b) Minimum Essential Medium (MEM; ThermoFisher Scientific, Fyrislund, Sweden) with 0.1 mM non-essential amino acids (Sigma-Aldrich, Stockholm, Sweden); SNU449, SNU398 and SNU423 in RPMI-1400 medium (Sigma-Aldrich, Stockholm, Sweden); PLC/PRF5, Huh7, HLF, HepG2, HaCaT and 293-T in Dulbecco’s Modified Earle Medium (Sigma-Aldrich, Stockholm, Sweden). All media contained 10% fetal bovine serum (FBS; Biowest, Esbjerg, Denmark), 100 U/ml penicillin, 100 μg/ml streptomycin and 2 mM glutamine (Sigma-Aldrich, Stockholm, Sweden).

HCC CAFs were isolated at the University of Vienna Hospital^22^. Patient consent and ethical approval were based on regulations of the Comprehensive Cancer Center Vienna. AKH12 were isolated from a patient with low-to-medium differentiated HCC, cirrhosis and vascular invasion (stage 3 by Edmondson and Steiner); AKH14 from a stage 2 HCC with focal fibrosis but no cirrhosis; AKH38 from a multifocal, fibrolamellar stage 2 HCC. None of the CAFs were infected by hepatitis-A, B, C or human immunodeficiency viruses^22^. CAFs were maintained in RPMI-1400, 5% FBS, 100 U/ml penicillin, 100 μg/ml streptomycin, 10 mM Hepes, 0.5 mM sodium pyruvate, 2 mg/ml bovine serum albumin (BSA), 10 μg/ml apotransferrin, 10 μM phosphoethanolamine, 10 μM ethanolamine, 25 nM sodium selenite, 50 nM hydrocortisone, 100 pM 3,3´,-5-triiodothyronine, 100 μg/ml insulin (Sigma-Aldrich, Stockholm, Sweden), and 1 ng/ml epidermal growth factor (PeproTech EC Ltd, London, UK). Cells were free of mycoplasma (tested every 2 months) and all established cell lines were authenticated using PCR-single-locus-technology (Eurofins, Uppsala, Sweden).

Cells were serum-deprived for 16 h; 5 ng/ml TGFβ1 (PeproTech EC Ltd, London, UK) in the absence or presence of 5 μM T0901317 or GW3965 (provided by Timothy C. Gahman and Andrew K. Shiau, Ludwig Cancer Research, La Jolla, CA, USA) were added at 80% confluency for 48 or 72 h, as specified. CAFs were stimulated with ligands without prior serum deprivation. Dimethylsulfoxide (DMSO), vehicle to T0901317 and GW3965, was added as control.

### Three-dimensional hepatosphere culture

Hep3b and SNU449 cells (1.5 × 10^3^ per well) were cultured alone or co-cultured with AKH12 CAFs (ratio 3:1 or 6:1 parenchymal cells:CAFs; both conditions resulted in similar hepatospheres), for 96 h in 10% FBS/MEM (Hep3b) or RPMI-1400 (SNU449), respectively. For hepatosphere formation, the 96-well plate GravityPLUS^TM^ Hanging Drop System (InSphero AG, Schlieren, Switzerland) was used according to the manufacturer´s instructions. Where indicated, 5 μM T0901317 was added separately on SNU449 and AKH12 cells for 24 h prior to sphere formation, in order to assess compound effects on a single population. LY2157299 (2 μM, Sigma-Aldrich, Stockholm, Sweden) was administered during sphere formation. Hepatospheres were photographed in the InSPHERO plates using a Zeiss Axioplan-2 microscope with 10 × objective lens, at ambient temperature and without immersion oil, via a Hamamatsu C4742-95 CCD digital camera and acquisition software Volocity® (PerkinElmer Waltham, MA, USA). Hepatospheres were assessed as the ratio of wells containing uniform spheres larger than 50 µm in diameter (cell aggregations of diverse and non-uniform shape were not analyzed), relative to the total number of wells. Sphere size was determined via Image J analysis (NIH, USA). Triplicate (*n*_*b*_ = 3) biological experiments were performed in 10 technical replicates (*n*_*t*_ = 10) per condition.

### Immunoblotting

CAFs (2 × 10^4^ cells per 12-well dish), Hep3b and SNU423 (5 × 10^5^ cells per 60 mm dish) and SNU398, SNU449, HLF, Huh7 and PLC/PRF5 (4 × 10^5^ cells per 60 mm dish), after the specified treatments, were washed in ice-cold phosphate buffer saline (PBS), pH 7.4, lysed and analyzed by immunoblot as described^20^, with antibodies at the following dilutions: fibronectin, 1:10,000 (Sigma-Aldrich, Stockholm, Sweden, F3648); fatty acid synthase (FASN), 1:1,000 (ab22759); calponin, 1:2,000 (EP798Y, ab46794); LXRα, 1:1,000 (ab41902); Smad3, 1:1,500 (ab40854), all from Abcam, Cambridge, United Kingdom; α-smooth muscle actin (αSMA), 1:500 (Santa Cruz Biotech Inc., Santa Cruz, CA, USA, sc1a4); GAPDH, 1:50,000 (Ambion, ThermoFisher Scientific, Fyrislund, Sweden, AM4300). Horseradish peroxidase-conjugated anti-mouse or anti-rabbit secondary antibodies (ThermoFisher Scientific, Fyrislund, Sweden) were used at 1:20,000 dilution. Triplicate (*n*_*b*_ = 3) biological experiments were performed in 2 technical replicates (*n*_*t*_ = 2) per condition. Densitometric quantification of protein bands was performed using the Fujifilm Intelligent Dark Box II program of a Fuji Aida digital scanner (Fujifilm Nordic AB, Stockholm, Sweden).

### Immunocytochemistry (ICC)

CAFs (1 × 10^4^ cells per 8-chamber well) were treated at 50% confluency with TGFβ1 (5 ng/ml) and/or T0901317 (5 μM) for 48 h prior to F-actin and αSMA detection, as described^20^. The antibodies used were: anti-αSMA (dilution 1:600, Santa Cruz Biotech Inc., Santa Cruz, CA, USA, sc1a4) in 1% BSA/PBS overnight (16 h) at 4 °C; anti-mouse Alexa Fluor-488 secondary antibody (1:1,000 in PBS; Invitrogen, ThermoFisher Scientific, Fyrislund, Sweden) for 1 h in the dark. Tetramethylrhodamine-isothiocyanate-phalloidin (dilution 1:1,000 in 1% BSA/PBS; Sigma-Aldrich, Stockholm, Sweden) staining lasted for 30 min at 24°C, and incubation with 4’,6-diamidino-2-phenylindole (DAPI, 1:1,000 in PBS; Sigma-Aldrich, Stockholm, Sweden) for 5 min was followed by three rinses in PBS. Triplicate (*n*_*b*_ = 3) biological experiments were performed in 2 technical replicates (*n*_*t*_ = 2) per condition.

### Molecular cloning

The human *αSMA/ACTA2* gene promoter was cloned from human immortalized keratinocyte HaCaT genomic DNA, using *hACTA2* promoter −1400/+50 bp-specific primers for PCR: Forward, 5´-AAAAACTCGAGTCAAACAGATCTGA CATAGTAACATGAGTGAACAGCTGGTCATGGC-3´; reverse, 5´- TTTTTAAGCTTCA GGGAAGCTGAAAGCTGAAGGGTTATATAGCCCCTTGG-3´. Amplified DNA was purified by agarose gel electrophoresis and digested by XhoI/HindIII and inserted into the pGL4.10-luciferase vector (Promega Corp., Stockholm, Sweden).

### Luciferase assay

HepG2 or AG1523 cells (1.8 × 10^4^ cells per 24-well) were transfected with luciferase-encoding together with pCMV-β-galactosidase plasmids (100 ng), the latter as reference, using Lipofectamine-3000 (Life Technologies, Stockholm, Sweden) for 48 h, and assayed as described^20^. The plasmids were: synthetic Smad-binding promoter CAGA_12_-luc^24^, −1400/+50 bp *hACTA2* promoter-luciferase, pCDNA3-Flag-Smad3/pCDNA3-Flag-Smad4^30^, and pCMX-LXRα^20^. Serum-starved cells were stimulated with TGFβ1 (5 ng/ml) for 16 h in the absence or presence of T0901317 (5 μM), GW3965 (5 μM) or their combination. Quadruplicate (*n*_*b*_ = 4) biological experiments were performed in 3 technical replicates (*n*_*t*_ = 3) per condition.

### DNA affinity precipitation (DNAp) assay

293-T (3 × 10^5^ per 6-well) and HepG2 (4 × 10^5^ per 60-mm dish) cells were transfected with pCMX-LXRα and/or pCDNA3-Flag-Smad3 plasmids (200 ng) for 48 h, using calcium phosphate (293-T) or lipofectamine-3000 (HepG2; Life Technologies, Stockholm, Sweden). Intact or transfected cells were lysed in 20 mM Tris, pH 7.5, 100 mM NaCl, 0.5% NP-40, 0.5 mM EDTA and protease inhibitor cocktail (Roche Diagnostics, Bromma, Sweden). Lysates were pre-cleared with protein-A beads (ThermoFisher Scientific, Fyrislund, Sweden), incubated with 0.9 μg biotinylated DNA probe (Eurofins, Uppsala, Sweden) and 15 μg salmon sperm DNA (in-house) for 90 min (293-T) or 3 h (HepG2), and with magnetic streptavidin-sepharose beads (GE Healthcare, Uppsala, Sweden) for 45 min; three washes were performed with lysis buffer prior to sequential immunoblot analysis on the same membrane using LXRα and Smad3 antibodies (see immunoblotting). The biotinylated double-stranded *hACTA2* promoter probe sequence was: 5’-CAAGGAGGTTAGTGGGCAGAGAGGAGGGCTACAGAGGC-3’. Quadruplicate (*n*_*b*_ = 4) biological experiments were performed in 2 technical replicates (*n*_*t*_ = 2) per condition.

### Chromatin immunoprecipitation (ChIP)

Cells were fixed in 2% formaldehyde for 10 min at 37 °C, washed in ice-cold PBS twice, scraped in PBS and centrifuged at 4000 rpm for 5 min. Cells were lysed in 1% SDS, 10 mM EDTA, 50 mM Tris, pH 8.1, with protease inhibitors, for 20 min on ice, and sonicated (250 bp average DNA size). Lysates were diluted 10 times in 0.01% SDS, 1.0% Triton X-100, 1.2 mM EDTA, 16.7 mM Tris-HCl, pH 8.1, 167 mM NaCl, with protease inhibitors, and immunoprecipitated using anti-Smad2/3 (610843, Becton Dickinson & Co, Franklin Lakes, NJ, USA) or control rabbit antiserum, overnight at 4 °C, followed by protein-A dynabead incubation for 2 h at 4°C, washing once in 1% Triton X-100, 2 mM EDTA, 20 mM Tris-HCl, pH 8.1, 150 mM NaCl, once in 0.1% SDS, 1% Triton X-100, 2 mM EDTA, 20 mM Tris-HCl, pH 8.1, 500 mM NaCl, once in 0.25 M LiCl, 1% IGEPAL, 1% deoxycholate, 1 mM EDTA, 10 mM Tris-HCl, pH 8.1, and twice in 10 mM Tris-HCl, pH 8.0, 1 mM EDTA. Beads and input samples were re-suspended in 1% SDS, 1 mM NaHCO_3_ for 30 min and de-crosslinked in the presence of 0.5 M NaCl at 65°C overnight. The chromatin was subjected to proteinase-K digestion followed by phenol-chloroform extraction. Respective input was used to normalize the DNA in each ChIP sample. PCR analysis followed with primers for: human plasminogen activator inhibitor 1 (PAI-1), forward, 5′-GCAGGACATCCGGGAGAGA-3′; reverse, 5′-CCAATAGCCTTGGCCTGAGA-3′; human ACTA2 (−1,368/−1,265 bp), forward, 5′-CAGCTGGTCATGGCTGTAAAATAAAG-3′; reverse, 5′-CTCATAAAGAAATATTTTTGTGGGTACTG-3′; human ACTA2 (−805/−685 bp), forward, 5′-CATATCACCTGGTCTCTCTACTTC-3′; reverse 5′-CCCAG CAGCCTGTCAAAGA-3′; human ACTA2 (−161/−71 bp), forward, 5′-CCGCCTCCCGTT TCATG-3′; reverse, 5′-GACCTCAGCACAAAACTCTC-3′. Triplicate (*n*_*b*_ = 3) biological experiments were performed in four technical replicates (*n*_*t*_ = 4) per condition.

### Reverse transcription and real-time qPCR

CAFs (2 × 10^4^ per 12-well), Hep3b and SNU423 (5 × 10^5^ per 60-mm dish), and SNU398, SNU449, HLF, Huh7 and PLC/PRF5 cells (4 × 10^5^ per 60-mm dish), were cultured for total RNA isolation using the NucleoSpin RNA II kit (Macherey Nagel, AH Diagnostics, Solna, Sweden), according to the manufacturer´s instructions. Reverse transcriptase real-time PCR was performed using primers (Table 1) as described^20^. Gene expression levels were normalized to the reference gene *GAPDH* and calculated as 2^−ΔCt^ (ΔC_t_ = C_ttest mRNA_ – C_tGAPDH mRNA_). At least three biological (*n*_*b*_ = 3) experiments, each in technical triplicate (*n*_*t*_ = 3) are described.

**Table 1:** Human primer sequences used for quantitative real-time PCR

GENE	Forward	Reverse
*GAPDH*	GGAGTCAACGGATTTGGTCGTA	GGAGTCAACGGATTTGGTCGTA
*LXRα*	CCACCGAGACTTCTGGACAGG	GCAGAGTCAGGAGGAATGTCAGG
*αSMA*	TTACTACTGCTGAGCGTGAGATT	CTTCTCAAGGGAGGATGAGGATG
*FASN*	AGCTGCCAGAGTCGGAGAAC	TGTAGCCCACGAGTGTCTCG
*SM22α*	GGTTTATGAAGAAAGCGCAGGAG	CTCTAACTGATGATCTGCCGAGG
*Calponin*	GAGGTTAAGAACAAGCTGGCCC	TTGATGAAGTTGCCGATGTTCTC

The table lists human gene names along with the sequences (5´–3´direction) of gene-specific primers used for PCR analysis.

### Statistical analysis

Experiments were performed in biological replicates (*n*_*b*_), most of which included at minimum technical triplicates (*n*_*t*_ = 3), as indicated in the figure legends. Sample size depended on the assay type: for sphere quantification assays a minimum of 25 spheres was measured per experiment to reach statistical power for discrimination between conditions. For all groups that are statistically compared, the variance within each group was very similar, close to identical. Representative results are reported as averages minus-plus standard deviation, s.d. Statistical analysis was performed with GraphPadPrism (La Jolla, CA, USA) using two-sided *t*-test, and statistical significance was assigned with *p-value < 0.05; **p < 0.01; ***p < 0.001.

## Supplementary information

Supplemental Figure

Figure S1
