# Supplementary material for: LXRα limits TGFβ-dependent hepatocellular carcinoma associated fibroblast differentiation
Source: Oncogenesis. 2019 May 16;8(6):36. doi: 10.1038/s41389-019-0140-4 (PMC6522550; doi:10.1038/s41389-019-0140-4)
Supplement: Supplementary file 1 — Supplemental Figure [file 41389_2019_140_MOESM1_ESM.docx]

**LXRα limits TGFβ-dependent hepatocellular carcinoma associated fibroblast differentiation**

Anita Morén, Claudia Bellomo, Yutaro Tsubakihara, Dimitris Kardassis, Wolfgang Mikulits, Carl-Henrik Heldin and Aristidis Moustakas

**Supplementary Information**

**Supplementary Figure legends**

**Supplementary Fig. S1.** **The LXRα agonist T0901317 signals by inducing *FASN* gene expression in HCCs.** The indicated human HCC cells were stimulated with DMSO (Mock), 5 ng/ml TGFβ1, 5 µM T0901317 or a combination of the last two for 72 h prior to cell lysis and analysis by real-time PCR for *FASN* normalized to the expression of *GAPDH*. Bars in the graphs are color-coded according to Figure 1. Mean±SD values are plotted. Experiments were performed in biological duplicates (n_b_=2), each of them in technical triplicate (n_t_=3). Statistical comparison (two-sided t-test) indicates significant differences, **p*<0.05, ***p*<0.01, ****p*<0.001, *****p*<0.0001, ns, *p*>0.05.
