# Supplementary figures and images for: LXRα limits TGFβ-dependent hepatocellular carcinoma associated fibroblast differentiation
Source: Oncogenesis. 2019 May 16;8(6):36. doi: 10.1038/s41389-019-0140-4 (PMC6522550; doi:10.1038/s41389-019-0140-4)

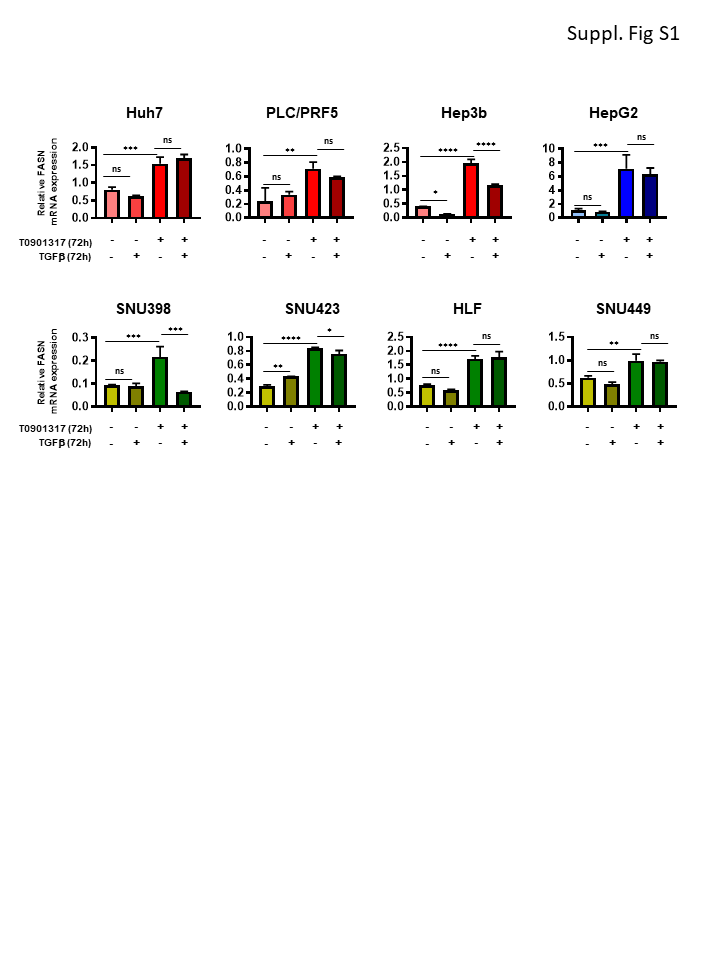

Supplement: Supplementary file 2 — Figure S1 [file 41389_2019_140_MOESM2_ESM.tif]
